# Serum Magnesium is associated with Carotid Atherosclerosis in patients with high cardiovascular risk (CORDIOPREV Study)

**DOI:** 10.1038/s41598-019-44322-z

**Published:** 2019-05-29

**Authors:** M. Encarnación Rodríguez-Ortiz, Francisco Gómez-Delgado, Antonio P. Arenas de Larriva, Antonio Canalejo, Purificación Gómez-Luna, Carmen Herencia, Javier López-Moreno, Mariano Rodríguez, José López-Miranda, Yolanda Almadén

**Affiliations:** 1 Instituto Maimónides de Investigación Biomédica de Córdoba (IMIBIC), Reina Sofia University Hospital/University of Cordoba, Cordoba, Spain; 2 Unidad de Gestión Clinica Medicina Interna. Lipid and Atherosclerosis Unit. Department of Internal Medicine/IMIBIC/Reina Sofia University Hospital/University of Cordoba, Cordoba, Spain; 3 CIBER Fisiopatologia Obesidad y Nutricion (CIBEROBN), Instituto de Salud Carlos III, Madrid, Spain; 4 Department of Integrated Sciences/Centro de investigacion RENSMA, University of Huelva, Huelva, Spain; 5 Renal, Vascular and Diabetes Research Laboratory, Fundación Instituto de Investigaciones Sanitarias-Fundación Jiménez Díaz, Universidad Autónoma de Madrid, Madrid, Spain; 6 Fuente Palmera Primary Health Care Center, Córdoba-Guadalquivir Health District, Córdoba, Spain

**Keywords:** Risk factors, Predictive markers

## Abstract

This study aimed to ascertain whether there is an independent association between serum magnesium (Mg) and the Carotid Intima-Media Thickness (IMT-CC), a well-accepted atherosclerotic-biomarker surrogate of cardiovascular disease (CVD), in a population with high cardiovascular risk. Serum Mg and traditional atherosclerotic risk factors were recorded in 939 patients (mean age, 59.6 ± 0.3 years, 83.2% men) with coronary heart disease (CHD) enrolled in the CORDIOPREV trial. Serum Mg strongly associated with IMT-CC. Before adjusting for potential confounding factors, IMT-CC decreased by 0.111 ± 0.011 mm per mg/dl increase in serum Mg (*p* < 0.001). After adjustment, the effect of Mg did not appear mediated through factors related to glucose metabolism, the lipid profile or the mineral metabolism and renal function. Multivariate models showed the lower Mg levels (quartile 1) as a strong independent factor contributing to IMT-CC along with age, sex, SBP, HDL-C, and diuretic use. Logistic regression analysis confirmed the predictive ability of serum Mg to differentiate patients at higher atherosclerotic risk as defined by an IMT-CC ≥ 1.0 mm, yielding a OR for the lower quartile of 10.623 (95%CI 2.311–48.845; P = 0.002) and a ROC-derived cutoff of 1.61 mg/dl. Therefore, our findings outline low serum magnesium as a possible independent risk factor for carotid atherosclerosis.

## Introduction

Cardiovascular disease (CVD) is considered one of the most important causes of mortality and morbidity around the world. Improvement in the risk factors has led to lower the incidence rates and thus to a decrease in mortality. However, traditional cardiovascular risk factors as aging, obesity, hypertension, diabetes, dyslipidemia, inflammation, etc., cannot explain a significant proportion of cases and thus, new risk factors are being on search^1^. In this regard, factors related to mineral metabolism, such as magnesium (Mg) concentration, might play a role in the development of cardiovascular disease (CVD)^2–5^.

Observational studies have shown an association between reduced serum levels of Mg and a number of CVD biomarkers and endpoints such as the risk of ischemic stroke^6,7^, high blood pressure^8^, dyslipidemia^9^, type 2 diabetes mellitus^9,10^ and mortality^11^. The mechanisms whereby low Mg may produce cardiovascular damage are not well defined but it is known that hypomagnesaemia is associated with endothelial dysfunction^5^ and increased production of pro-inflammatory cytokines and neuropeptides^12^, may increase the incidence of ischemic stroke via effects on hypertension and diabetes^7^ and may cause vascular calcification^13^. Conversely, epidemiologic, prospective and meta-analysis studies have favourably associated Mg with a decreased risk of CVD^2,4,14,15^. High dietary Mg and moderate hypermagnesaemia had a protective effect on endothelial dysfunction, insulin resistance, vascular calcification, inflammation, and atherosclerosis^2,16,17^. Dietary magnesium intake was also associated with reduced mortality from CVD^18^. Of note, the administration of Mg improved endothelial function in patients with coronary artery disease, especially in those with the lowest intracellular Mg content^19^. However, surveys and studies have shown that dietary magnesium intake is often inadequate in the western countries and hypomagnesaemia is often underdiagnosed in hospitalized patients^14^. Therefore, though further research into the effectiveness of Mg supplementation for individuals at higher risk of CVD is needed, some scientific agencies have established recommendations on Mg intake to prevent CVD^20^.

Atherosclerosis is a key cause of CVD that can be conveniently monitored by non-invasive imaging techniques, such as high resolution B-mode ultrasound, to be detected and quantified in terms of intima-media thickness of both common carotid arteries (IMT-CC)^21^. Therefore, as a good surrogate marker of subclinical atherosclerosis, the IMT-CC predicts the prognosis of CVD and is a strong predictor of future vascular events^22,23^. Previous studies found an inverse relationship between Mg levels and the carotid IMT-CC scores in the general population^9,24,25^ and haemodialysis patients^26^, but further studies are warranted to elucidate this effect in a population with high risk of CVD. In addition, ethnic and environmental factors may modify the Mg-atherosclerosis associations^9,25^. Thus, the aim of the present cross-sectional study was to determine whether there is a relationship between serum Mg level and the IMT-CC in a large cohort of European patients with high cardiovascular risk diagnosed of CHD but with preserved renal function.

## Results

This cross-sectional analysis included 939 patients. The mean age was 59.6 ± 0.3 and 83.2% were men. Anthropometric characteristics, medication and variables of glucose metabolism, lipid profile, mineral metabolism and renal function are presented in Table 1 separated by quartiles of serum Mg concentrations. Lower quartiles of serum Mg were associated with increased IMT-CC, and also with age, SBP, fasting glucose, glycated hemoglobin (HbA1c), HOMA-IR, serum corrected calcium, diabetes, oral antidiabetic drugs and insulin use. Conversely, higher quartiles of Mg concentration were associated with, serum HOMA-Beta, HDL-C, TC, LDL-C, ApoA-I and eGFR.

**Table 1:** Baseline characteristics of the participants according to quartiles of magnesium.

	Quartiles of Magnesium				P value
	Quartile 1 (Low)	Quartile 2	Quartile 3	Quartile 4 (High)	
Magnesium (mg/dl)	1.29 ± 0.02	1.74 ± 0.01	1.96 ± 0.04	2.31 ± 0.01	<0.001
Age (years)	62.4 ± 0.6	59.4 ± 0.6	58.4 ± 0.6	57.8 ± 0.6	<0.001
Sex (%Men)	82.8	81.1	85.3	85.7	0.494
Weight (Kg)	85.3 ± 0.9	84.5 ± 0.9	84.1 ± 0.9	85.4 ± 0.9	0.913
BMI (Kg/m^2^)	31.3 ± 0.3	30.9 ± 0.3	30.7 ± 0.3	31.0 ± 0.3	0.470
Waist circumference (cm)	106.7 ± 0.7	104.8 ± 0.7	104.4 ± 0.8	104.7 ± 0.8	0.123
DBP (mmHg)	77.3 ± 0.7	76.2 ± 0.7	77.8 ± 0.7	77.3 ± 0.7	0.484
SBP (mmHg)	145.5 ± 1.3	137.6 ± 1.3	135.8 ± 1.3	135.2 ± 1.3	<0.001
Fasting Glucose (mg/dl)	128.2 ± 2.7	113.7 ± 2.7	109.6 ± 2.8	106.4 ± 2.7	<0.001
HbA1c (%)	7.15 ± 0.07	6.61 ± 0.07	6.52 ± 0.07	6.25 ± 0.07	<0.001
Fasting Insulin(mU/L)	12.4 ± 0.7	11.0 ± 0.7	9.89 ± 0.7	10.6 ± 0.7	0.089
HOMA-IR	5.12 ± 0.28	4.19 ± 0.29	3.63 ± 0.30	3.83 ± 0.29	0.001
HOMA-Beta	75.3 ± 3.5	82.9 ± 3.5	86.7 ± 3.6	93.4 ± 3.5	0.003
HDL-C (mg/dl)	40.7 ± 0.6	42.3 ± 0.6	42.0 ± 0.7	43.8 ± 0.7	0.011
Total cholesterol (mg/dl)	153.7 ± 2.1	159.6 ± 2.1	158.9 ± 2.1	165.4 ± 2.1	0.002
Tryglicerides (mg/dl)	140.5 ± 5.8	137.8 ± 5.9	132.5 ± 6.0	150.7 ± 5.9	0.183
LDL-C (mg/dl)	84.2 ± 1.7	89.0 ± 1.7	90.5 ± 1.8	91.0 ± 1.8	0.020
ApoA-I (mg/dl)	125.9 ± 1.4	129.9 ± 1.4	128.5 ± 1.4	134.5 ± 1.4	<0.001
ApoB (mg/dl)	72.8 ± 1.2	73.3 ± 1.2	73.2 ± 1.2	75.9 ± 1.2	0.267
Creatinine (mg/dl)	0.92 ± 0.01	0.88 ± 0.01	0.87 ± 0.01	0.89 ± 0.01	0.095
eGFR (ml/min)	89.3 ± 1.3	93.1 ± 1.3	94.7 ± 1.4	94.8 ± 1.4	0.012
Phosphate(mg/dl)	3.62 ± 0.04	3.64 ± 0.04	3.58 ± 0.05	3.53 ± 0.05	0.318
Corrected calcium (mg/dl)	9.64 ± 0.03	9.55 ± 0.03	9.52 ± 0.03	9.53 ± 0.03	0.010
IMT-CC (mm)	0.81 ± 0.01	0.70 ± 0.01	0.68 ± 0.01	0.68 ± 0.01	<0.001
Smoking (% current smokers)	10.1	8.90	9.00	9.70	0.965
Diabetes (%)	64.1	35.2	31.6	13.0	<0.001
**Medication use**					
Lipid lowering drugs:					
Statins (%)	87.3	86.4	87.2	81.5	0.234
Fibrates (%)	0.80	2.10	0.90	1.80	0.593
Others (%)	4.60	4.70	5.70	4.80	0.952
Diuretic use	40.9	39.0	36.4	35.7	0.615
Nitrates	10.7	8.1	8.2	8.7	0.722
Antiarrhythmic drug	2.0	3.3	1.3	2.1	0.544
Oral anticoagulant drugs	2.8	2.4	1.7	1.7	0.794
Proton Bomb Inhibitors	75.4	79.3	76.6	76.3	0.765
Antidepressant	9.9	8.9	9.5	12.0	0.694
Oral Antidiabetic drugs	22.2	10.2	11.3	5.4	<0.001
Insulin use	14.7	6.5	5.6	3.3	<0.001

Values are means ± SE. Continuous variables were compared using the analysis of variance (ANOVA). Qualitative variables were compared using Chi Square test. BMI, body mass index; HbA1c, hemoglobin A1c; HOMA-IR, Homeostasis Model Assessment-Insulin Resistance; HOMA-Beta, Homeostasis Model Assessment-beta cell function; HDL-C, high-density lipoprotein-cholesterol; LDL-C, low density lipoprotein-cholesterol; ApoA-I, apoliprotein A-I; ApoB, Apolipoprotein B; eGFR, estimated glomerular filtration rate.

**Figure 1:**
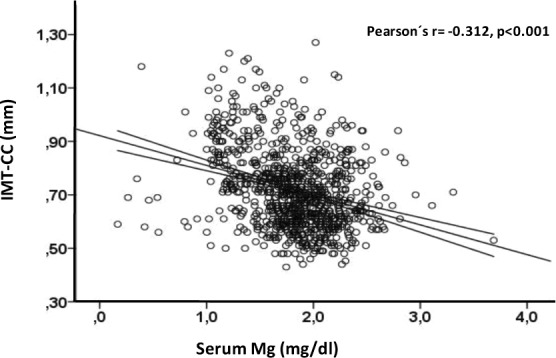
The scatter plot between continuous serum Mg and IMT-CC values. Regression line and 95% confidence intervals are shown.

As shown in Table 2 and Fig. 1, serum Mg was highly correlated with IMT-CC (Pearson´r = −0.312, p < 0.001). Among anthropometric parameters, age, gender, SBP and waist circumference, but not BMI, were correlated with IMT-CC values. A positive association was also found for the presence of diabetes and two medications as insulin and diuretic use. Regarding parameters of glucose metabolism, IMT-CC was positively correlated with diabetes, serum glucose and HbA1c and negatively correlated with HOMA-Beta. In relation to the lipid profile, IMT-CC exhibited a significant, negative correlation with HDL-C and ApoA-I. Lastly, among parameters of mineral metabolism and renal function, IMT-CC correlated negatively with serum Mg concentration and eGFR and positively with serum creatinine.

**Table 2:** Univariate analysis (correlation coefficients) between study variables and IMT-CC scores.

	Correlation coefficients	P value	Type of Coefficient
Magnesium (mg/dl)	−0.312	<0.001	r
Age (years)	0.347	<0.001	r
Sex	−0.106	0.001	rho
Weight (Kg)	0.044	0.183	r
BMI (Kg/m^2^)	0.048	0.139	r
Waist circumference (cm)	0.144	<0.001	r
DBP (mmHg)	−0.024	0.457	r
SBP (mmHg)	0.226	<0.001	r
Fasting Glucose (mg/dl)	0.103	0.002	r
HbA1c (%)	0.184	<0.001	r
Insulin (mU/L)	0.019	0.568	r
HOMA-Beta	−0.073	0.030	r
HOMA-IR	0.057	0.082	r
HDL-C (mg/dl)	−0.108	0.001	r
Total cholesterol (mg/dl)	−0.034	0.306	r
Tryglicerides (mg/dl)	0.018	0.581	r
LDL-C (mg/dl)	−0.034	0.308	r
ApoA-I (mg/dl)	−0.114	0.001	r
ApoB (mg/dl)	0.020	0.543	r
Creatinine (mg/dl)	0.134	<0.001	r
eGFR (ml/min)	−0.135	<0.001	r
Phosphate (mg/dl)	0.046	0.174	r
Corrected calcium (mg/dl)	0.028	0.405	r
Smoking (current smoker: yes/no)	0.017	0.601	rho
Diabetes (yes/no)	0.170	<0.001	rho
**Medication use**			
Lipids lowering drugs:			
Statins (yes/no)	0.050	0.124	rho
Fibrates (yes/no)	−0.038	0.239	rho
Others (yes/no)	−0.015	0.653	rho
Diuretic use (yes/no)	0.110	0.001	rho
Nitrates (yes/no)	0.005	0.881	rho
Antiarrhythmic drug (yes/no)	0.004	0.915	rho
Oral anticoagulant drugs (yes/no)	0.052	0.113	rho
Proton Bomb Inhibitors (yes/no)	0.009	0.783	rho
Antidepressant (yes/no)	−0.017	0.605	rho
Oral Antidiabetic drugs (yes/no)	0.061	0.062	rho
Insulin use (yes/no)	0.073	0.025	rho

The Pearson (r) coefficient or the Spearman (rho) coefficient is shown for continuous or qualitative variables, respectively. BMI, body mass index; HbA1c, hemoglobin A1c; HOMA-IR, Homeostasis Model Assessment-Insulin Resistance; HOMA-Beta, Homeostasis Model Assessment-beta cell function; HDL-C, high-density lipoprotein-cholesterol; LDL-C, low density lipoprotein-cholesterol; ApoA-I, apoliprotein A-I; ApoB, Apolipoprotein B; eGFR, estimated glomerular filtration rate.

To separate the effect of Mg from that of the previously determined significant CVD risk factors, multiple linear regression analysis with IMT-CC as the dependent variable was applied. By considering serum Mg as a continuous variable, before adjusting for potential confounding factors, IMT-CC decreased by 0.111 ± 0.011 mm per mg/dl increase in serum Mg (*p* < 0.001). After categorizing serum Mg by quartiles (Table 3), using quartile 4 (the higher levels) as the reference, only quartile 1 resulted significant throughout all the models. After adjusting for anthropometric factors (age, sex, waist circumference and SBP) and Medication (insulin and diuretic use) included in Model 1, the relationship of IMT-CC with Mg was attenuated but remained highly significant. After controlling only for parameters associated with glucose metabolism in Model 2 (glucose, HbA1c, HOMA-Beta and diabetes), the contribution of Mg to IMT-CC scores decreased slightly but remained highly significant. Similarly, the effect of Mg on IMT-CC was insignificantly modified after correction for CVD risk factors related to the lipid profile (Model 3: HDL-C and ApoA-I) or to the renal function (Model 4: eGFR and serum creatinine).A further adjustment for anthropometric factors and medication in Models 2, 3 and 4 (mixed Models 1 + 2, 1 + 3 and 1 + 4) yield results similar to those observed for the Model 1 alone.

**Table 3:** Association between IMT-CC and the lower serum Mg levels (quartile 1).

Independent variables	No standardized coefficients		Standardized coefficients	P value
	B	Standard error	Beta	
Unadjusted	0.124	0.010	0.373	<0.001
Model 1	0.098	0.010	0.296	<0.001
Model 2	0.115	0.011	0.344	<0.001
Model 3	0.121	0.010	0.362	<0.001
Model 4	0.123	0.010	0.367	<0.001
Model 1 + 2	0.099	0.011	0.294	<0.001
Model 1 + 3	0.097	0.010	0.291	<0.001
Model 1 + 4	0.100	0.010	0.299	<0.001

Serum Mg levels were categorized by quartiles. Quartile 4 (higher serum Mg levels) was the reference group. Quartiles 2 and 3 showed no statistical significance. Adjustment for: Model 1: anthropometric factors (age, sex, waist circumference and SBP) and medication (insulin and diuretic use); Model 2: Factors related to glucose metabolism (glucose, HbA1c, HOMA-Beta and diabetes); Model 3: Factors related to lipid profile (HDL-C and ApoA-I); Model 4: Factors related to mineral metabolism and renal function (eGFR and serum creatinine). Model 1 + 2: Model 1 plus factors related to glucose metabolism. Model 1 + 3: Model 1 plus factors related to lipid profile. Model 1 + 4: Model 1 plus factors related to mineral metabolism. HDL-C, high-density lipoprotein-cholesterol; ApoA-I, apoliprotein A-I; HbA1c, hemoglobin A1c; HOMA-Beta, Homeostasis Model Assessment-beta cell function; eGFR, estimated glomerular filtration rate.

To determine independent predictors of IMT-CC score, a multivariable stepwise linear regression analysis was performed including all the risk factors that reached significance in the univariate analysis. As a continuous variable, serum Mg resulted a strong independent factor contributing to IMT-CC (−0.082 ± 0.012 mm per mg/dl, *p* < 0.001). Furthermore, as shown in Table 4, after categorizing for quartiles of serum Mg, the lower Mg levels (quartile 1) (*p* < 0.001), age (*p* < 0.001), sex (*p* < 0.001), SBP (*p* < 0.001), HDL-C (*p* = 0.016) and diuretic use (*p* = 0.031) were independent risk factors for IMT-CC levels. The serum Mg concentration (quartile 1) positively associated with IMT-CC with a marked contribution (standardized Beta 0.288, *p* < 0.001). A positive association was also observed for age, SBP and diuretic use, while a negative association was found for female sex and HDL-C. The predictive ability of serum Mg to distinguish those CHD patients at higher risk was also tested by a multiple logistic regression analysis using two different thresholds. Firstly, when it was defined as having a IMT-CC ≥ 0.7 mm, the mean value found in healthy middle-age adults^27,28^, only the lower serum Mg levels (quartile 1) (OR, 4.151; 95%CI 2.545–6.768; P < 0.001), age (OR, 1.069; 95%CI 1.046–1.091; P < 0.001), female sex (OR, 0.554; 95%CI 0.308–0.995; P = 0.048) and SBP (OR, 1.014; 95%CI 1.005–1.023; P = 0.001) appeared as significant predictors variables. Secondly, and taking into account that our study population is at high risk of CVD, we also considered the more severe threshold of IMT-CC ≥ 1.0 mm. It is of note that in this case, only the lower serum Mg (quartile 1) remained as a significant predictor variable (OR, 10.623; 95%CI 2.311–48.845; P = 0.002). In addition, the optimal cutoff values of serum magnesium levels to predict the risk were calculated from the ROC corresponding to each threshold. The AUC for the IMT-CC ≥ 1.0 mm ROC was 0.765 ± 0.034, 95% CI 0.697–0.832, and the cutoff 1.61 mg/dl, (more convenient than the AUC of 0.619 ± 0.020, 95% CI 0.615–0.683 and a cutoff of 1.79 mg/dl, corresponding to the IMT-CC ≥ 0.7 mm).

**Table 4:** Statistically significant IMT-CC score determinants and coefficients for stepwise multivariable linear regression analysis.

Independent variables	No standardized coefficients		Standardized coefficients	P value
	B	Standard error	Beta	
Serum Magnesium-Quartile 1	0.097	0.011	−0.288	<0.001
Age (years)	0.004	0.001	0.257	<0.001
Sex (female)	−0.060	0.013	−0.145	<0.001
SBP (mmHg)	0.001	0.000	0.122	<0.001
HDL-C (mg/dl)	−0.001	0.000	−0.077	0.016
Diuretic use (yes)	0.021	0.010	0.069	0.031

Predictive variables tested (independent variables that reached significance in the univariate associations)**:** serum Mg categorized by quartiles (quartile 4 as reference group), age, sex, waist circumference, SBP, diuretic use, fasting glucose, HbA1c, HOMA-Beta, HDL-C, ApoA-I, eGFR, and serum creatinine. Constant = 0.420; R^2^ = 0.253. HDL-C, high-density lipoprotein-cholesterol; ApoA-I, apoliprotein A-I; HbA1c, hemoglobin A1c; HOMA-Beta, Homeostasis Model Assessment-beta cell function; eGFR, estimated glomerular filtration rate.

## Discussion

Increasing evidences show that traditional risk factors fail to explain all the risk for CVD, which has lead to the search of new or emerging risk factors^1^. In the context of a randomized trial enrolling patients with CHD, the present study was conducted at base-line to ascertain an independent association between serum Mg and IMT-CC, a marker of carotid atherosclerotic vascular disease, in a population with high cardiovascular risk. The results indicated that higher values of IMT-CC were associated with the lower levels of serum Mg concentration. This inverse association was independent from traditional CVD risk factors such as abnormalities of lipid and glucose metabolism; though it was attenuated after adjusting for anthropometric factors as sex, age, waist perimeter and SBP and medication (insulin and diuretic use), still it remained highly significant.

Our results in a European population with CVD were consistent with previous cohort studies performed in the general population where an inverse association between serum Mg and IMT-CC had been described in American and Asian populations^9,24,25^. Interestingly, in a population without CHD, an inverse correlation between serum Mg and the relative risk of CHD^29^ and the incidence of stroke^6,7^ was also reported. Moreover, the intake of dietary Mg was associated with a reduced risk of CHD^15,30^.

It was previously shown that low Mg associated with higher fasting insulin levels^10^ and insulin resistance^31^. Previous data from the CORDIOPREV study also showed that glucose metabolism control was significantly associated with carotid atherosclerosis^32^. In our study, the presence of diabetes and the use of insulin were positively associated with IMT-CC; though fasting insulin did not. Besides, a significant positive association was observed for glucose and HbA1c and negative for HOMA-Beta. HbA1c was previously shown to be independently associated with IMT-CC in non-diabetic populations^33^. After adjusting for these factors, we observed only a modest decrease in the effect of serum Mg on IMT-CC. Therefore, these data reveals that the effect of Mg on IMT-CC cannot be explained by its association with factors related to glucose metabolism.

We found that serum Mg concentration was positively associated with CVD risk factors related to the lipid profile. Though some previous studies have found significant associations between serum Mg and the lipid profile^9,34^, others have not^26,35^. Furthermore, higher levels of Mg were associated with lower IMT-CC in a Chinese middle-age and elderly community^25^; and of note, the specific effect of Mg was decreased after adjusting for TC, HDL-C, and TG. However, the possible mediation of the lipid profile on the effect of Mg resulted unlikely because higher Mg levels were also associated with higher serum TC and LDL-C. In our study, HDL-C was inversely related to IMT-CC; while ApoA-I was positively associated. Of note, the relationship between IMT-CC and serum Mg was not altered after controlling for these. Thus, it appears that the effect of Mg is not significantly mediated through the lipid factors.

In patients with chronic kidney disease and in haemodialysis, serum Mg was negatively associated with IMT-CC, arterial stiffness and vascular calcification^26,36^. In the present study we found that, apart from serum Mg, none of the mineral metabolism parameters correlated with IMT-CC. Regarding the renal function, the eGFR positively associated with serum Mg and inversely associated with IMT-CC, whereas serum creatinine was the only variable positively associated with IMT-CC but it did not correlate with serum Mg. The contribution of serum Mg into IMT-CC did not change after adjusting for these parameters.

By taking into account simultaneously all these underlying confounding factors, multiple regression analysis revealed that low serum Mg concentration was a strong independent factor contributing to the increase of IMT-CC. It was accompanied by other well recognized atherosclerotic risk factors. Thus, a negative association was observed for female sex, while a positive association was observed for age and SBP. HDL-C appeared also as an independent factor negatively related to IMT-CC, which had been previously observed and related to its antioxidant activitiy^37^. We also observed the use of diuretics as an independent positive contributor though it did not correlated with serum Mg, which does not agree with the findings reported in the Atherosclerosis Risk in Communities Study (ARIC) cohort reporting that higher serum Mg levels associated to lower prevalence of diuretic use^7^. It is of note that, although taken as a continuous variable serum Mg showed a lineal relationship with IMT-CC, after categorizing by quartiles only that corresponding to the lower levels (quartile 1) resulted significant. Therefore, we can only state that the lower levels of serum Mg are associated with the higher levels of IMT-CC. Logistic regression models provided a good picture of the extent of this relationship. After controlling for the other covariates, along with other factors the lower quartile of serum Mg appeared as a significant predictor with a odds ratio of 4.151 to distinguish those CHD patients at higher risk defined as IMT-CC ≥ 0.7 mm^27,28^. But interestingly, after defining the risk with a cutoff as IMT-CC ≥ 1.0, more appropriated for a population at high CV risk, only the lower quartile of serum Mg remained as a significant predictor with an odds ratio of 10.623. Furthermore, in these conditions the AUC for the ROC was 0.765, yielding an optimal cutoff value of serum magnesium level of 1.61 mg/dl to discriminate well the patients at higher risk of atherosclerosis.

In an attempt to hypothesize potential biological mechanisms underlying the independent effect of Mg on the modulation of IMT-CC, it is interesting to consider that Mg appears related specifically with some key pathophysiological processes occurring at the vascular wall. One is vascular calcification^16,36^, which is negatively regulated through the Mg transport into the VSMCs, leading to Wnt/β-catenin-dependent down-regulation of Cbfa-1 and osterix to prevent its osteogenic differentiation^38,39^. In addition, hypomagnesaemia associates with endothelial dysfunction, a key early contributor to atherosclerosis, through inducing a number of effects on the endothelial cells such as an elevation of LDL transport and its oxidation and the release of cytokines and adhesion molecules; thus resulting in the instauration of a pro-inflammatory and pro-atherogenic setting^36,40^.

Though further work is needed to ascertain the specific mechanisms, our results support previous evidence pointing to magnesium deficiency as a missing link between diverse cardiovascular risk factors and atherosclerosis^2^. Since serum Mg levels appear as a predictive tool to identify those CHD patients at highest risk of CVD, it could be therefore useful to monitor serum Mg to avoid hypomagnesaemia in these patients. Indeed, it has been proposed that as an inexpensive and rather safe element, Mg could be useful in preventing and treating atherosclerosis^41^. However, Mg levels tend to be maintained remarkably constant, at least in healthy individuals, so that serum Mg is more likely to reflect its renal handling rather than its dietary intake. This can partially explain the differences found in studies addressing the effect of Mg intake, which was able to reduce the risk of CHD^15,30^ but was not associated to IMT-CC^9^. Thus, the convenience for Mg supplementation is unclear and remains to be fully elucidated.

Our study shows an independent effect of serum Mg on IMT-CC scores but we cannot establish any causal relationship. Serum Mg has been negatively associated to a number of important CV risk factors as obesity, hypertension, diabetes, dyslipidemia or inflammation^8–10,12^, which lead to questioning whether it might really have a separated effect or it is a mere biomarker of other risk factors. The elucidation of this issue is being quite elusive due to the controversial results obtained at evaluating the effect of Mg intake on different CV diseases in prospective studies, in part because Mg intake cannot easily be related to serum levels. A meta-analysis showed that while circulating Mg was associated with a 30% lower risk of CVD, dietary Mg was not^4^. On the other hand, reverse causation would also be a possibility that is being increasingly taken into account at evaluating associations of CV risk factors in epidemiological research^42^. Hence, the possibility that the association between the serum Mg and the IMT-CC severity might be derived from a pre-existing subclinical disease or lifestyle behaviors cannot be ruled out. It would be possible that any subtle derangement led to lower serum Mg concentrations. The prevalent CHD history or some underscored life style pattern related to the disease might result in increased IMT-CC levels and then to lower serum Mg concentrations. It is known that diabetes is accompanied by increased Mg excretion and insulin resistance decreases Mg uptake. Toward this uncertainty, genetic studies emerge as a powerful tool to uncover reverse causality^42^. In this line, it is notable that a couple of recent meta-analyses have addressed the causal association of serum Mg with CVD through Mendelian randomization studies using genetic variants predisposing to higher serum magnesium levels. The results provide evidence that genetically higher serum magnesium concentrations are associated with a reduced risk of CAD^43^ and cardioembolic stroke^44^, which gives a strong support to the role of serum Mg as an actual risk factor by its own.

There were limitations deserve considerations in this study. First, since it is a cross-sectional study, causal association between serum Mg and IMT-CC cannot be inferred; especially taking into account the dynamic nature of serum magnesium levels. Second, participants were recruited from a cardiovascular risk population under medical care; therefore, results cannot be extrapolated to healthy persons.

In conclusion, this study demonstrated that in patients with high cardiovascular risk, the lower levels of serum Mg were associated with an increase of IMT-CC, a well-accepted atherosclerotic-biomarker surrogate of CVD. This effect was independent from other traditional cardiovascular risk factors. Additional large interventional studies are needed to clarify causal relationship and whether Mg supplementation might be beneficial.

## Methods

### Subjects

This work was performed under the CORDIOPREV study (Clinical Trials Registry NCT00924937; URL: https://clinicaltrials.gov/ct2/show/NCT00924937. First registration: 19/06/2009). The CORDIOPREV study is a prospective, randomized, controlled trial that includes 1,002 patients with coronary heart disease (CHD), who had their last coronary event more than six months before joining the study. The objective of this study is to compare the ability of a Mediterranean diet rich in virgin olive oil versus a low-fat diet to influence the composite incidence of cardiovascular events after 7 years in subjects with documented CHD at baseline. Recruitment of patients lasted from November 2009 to February 2012, mostly at the Reina Sofia University Hospital (Cordoba, Spain). The inclusion and exclusion criteria have been published elsewhere^45^. Briefly, patients were 20–75 years old, with established CHD but without clinical events during the last six months, committed to follow a long-term monitoring study, with no other serious illnesses and a life expectancy of more than five years. From the initial sample of 1,002 subjects, in this article we included only the 939 subjects in whom carotid ultrasound was available together with baseline analytical and anthropometric data. The causes of the absence of data for the remaining 63 patients were as follows: 37 refused to conduct the echography, 14 withdrew from the study before conducting the tests, 12 other causes.

This study was conducted according to the ethical guidelines of the 1975 Declaration of Helsinki. The study protocol was approved by the Human Investigation Review Committee of the Reina Sofia University Hospital, according to institutional and Good Clinical Practice guidelines and participants gave their informed consent in writing to join the study.

### Risk factors assessment

After a 12-h fasting, patients were admitted to the clinical research facilities at 8.00 am for anthropometric measurements (weight, height, waist circumference and BMI). Patients had abstained from alcohol intake during the preceding 7 days and refrained from smoking during the fasting period. Venous blood was obtained from the antecubital vein and collected into Vacutainer tubes without anticoagulant and into EDTA containing tubes, and immediately transferred to 4 °C. Proteolytic degradation was minimized by supplementing plasma with 40 µl/ml of protease inhibitor cocktail (Roche Diagnostic, Germany). Until further biochemical analysis, Plasma and serum samples were frozen at −80 °C. Architect c-16000 analyzers (Abbott®,Chicago, IL, USA) were used to measure serum parameters by spectrophotometric techniques (enzymatic colorimetric methods): hexokinase method for glucose, and oxidation–peroxidation for HDL-C, TC and TG. LDL-C was calculated using the Friedewald formula (provided the TG level was below 400 mg/dl). ApoA-I and apoB were determined by immunoturbidimetry. Plasma levels of insulin were measured by chemiluminescent microparticle immunoassay using an analyzer (i-2000Abbott Architect®, Chicago, IL, USA). HbA1c was determined by HPLC. The insulin resistance (HOMA-IR) was calculated as (fasting insulin (µU/ml) × fasting glucose (mmol/l))/22.5. The beta-cell function (HOMA-Beta) was calculated as (fasting plasma insulin concentration (mU/l) × 20/(fasting plasma glucose (mmol/L) − 3.5)). Plasma creatinine, Ca, P and Mg were measured by spectrophotometry (BioSystems SA, Barcelona, Spain). eGFR was analyzed according to MDRD formula. Corrected calcium was calculated as (CaTotal/((ProteinTotal/18,5) + 0.6). A number of co-morbidities (as blood pressure, smoking status and presence of diabetes) and medications (i.e. Lipid lowering drugs, Diuretic use, Nitrates, Antiarrhythmic drug, Oral anticoagulant drugs, Proton Bomb Inhibitors, Antidepressant, Oral Antidiabetic drugs and Insulin use) were scored and used in this study for analysis.

### Ultrasound measurements of carotid artery wall

All patients were examined in supine position with their neck hyper-extended and their chin turned to the side. A Doppler ultrasound high-resolution B-mode (Envisor C Ultrasound System, Phillips, USA) was used to examine both carotid arteries following the recommendations of the American Society of Echocardiography Carotid Intima-Media Thickness Task Force^46^. Operators were unaware of the patients demographic and cardiovascular risk data. A semi-automatic software (QLAB Advance Ultrasound Quantification Software,v 5.0, Phillips, USA)) was used to obtain the measurements. For each patient, three measurements were taken to obtain the general mean of the intima-media thickness of both common carotid arteries (IMT-CC). Reliability estimates for IMT-CC measurements were 5.0 ± 2.6% and 5.13 ± 3.1%, for intra- and inter-observer variability coefficients (±standard deviation), respectively.

### Statistical analyses

Values of cardiovascular risk factors were analyzed in patients separated into quartiles of serum Mg. Analysis of variance was used to compare mean values of continuous measurements across quartiles. Categorical variables were presented as percentage and the differences were assessed using the χ^2^. Associations between IMT-CC scores and continuous or categorical variables were estimated using Pearson´s correlation or the Spearman rho coefficients, respectively. Multivariate models were used to estimate regression coefficient between the serum Mg (as a continuous variable or after categorizing for quartiles by using dummy variables) and the IMT-CC after adjusting for potential covariates (those that achieved significance at the univariate correlation) aggregated in different models. Model 1: anthropometric factors (age, sex, waist circumference and SBP) and medication (insulin and diuretic use); Model 2: Factors related to glucose metabolism (glucose, HbA1c, HOMA-Beta and diabetes); Model 3: Factors related to lipid profile (HDL-C and ApoA-I); Model 4: Factors related to mineral metabolism and renal function (eGFR and serum creatinine). To determine independent contributors of IMT-CC, a stepwise multivariate linear regression analysis (entry and stay significance levels of 0.05 and 0.10, respectively) was conducted with all the above significant factors. Multivariate logistic regression analysis was further performed to identify predictor variables for the atherosclerotic risk, as defined by an IMT-CC ≥ 0.7 mm or IMT-CC ≥ 1.0 mm. After constructing the corresponding ROCs, the cutoff value of serum Mg was also calculated based on the Youden’s index (J statistic) method. All analyses were performed using SPSS Statistics (version 20.0, SPSS Inc, Chicago, IL). A two-sided P-value of less than 0.05 was considered statistically significant.

## Data Availability

The data in this study are available from the corresponding authors on reasonable request.
